# The significance and prognostic value of multifocal papillary thyroid carcinoma in children and adolescents

**DOI:** 10.1186/s12885-024-12403-6

**Published:** 2024-06-06

**Authors:** Yuxiao Sun, Yihao Liu, Hongqiang Li, Yifeng Tang, Weihao Liu, Yifei Zhang, Detao Yin

**Affiliations:** 1 Department of Thyroid Surgery, The First Affiliated Hospital of Zhengzhou University, Zhengzhou, 450052 China; 2 Engineering Research Center of Multidisciplinary Diagnosis and Treatment of Thyroid Cancer of Henan Province, Zhengzhou, 450052 China; 3 Key Medicine Laboratory of Thyroid Cancer of Henan Province, Zhengzhou, 450052 China

**Keywords:** Papillary thyroid carcinoma, Children and adolescents, Persistent/recurrent disease, Multifocality

## Abstract

**Introduction:**

The prognostic value of multifocality in paediatric papillary thyroid carcinoma (PTC) patients remains a subject of debate. This study aimed to explore the clinical significance and prognostic value of multifocality in children and adolescents with PTC.

**Methods:**

This study retrospectively analysed the clinicopathological characteristics and postoperative follow-up data of 338 PTC patients aged ≤ 20 years from May 2012 to July 2022. The clinical and pathological characteristics of 205 patients with unifocal lesions and 133 patients with multifocal lesions were compared. A logistic regression model evaluated the relationship between multifocal lesions and disease recurrence/persistence in children and adolescents with PTC. Based on the median follow-up time of children with multifocal PTC, 114 patients with multifocal PTC older than 20 years were added, and the clinicopathological characteristics were compared between the 133. paediatric/adolescent patients and 114 adult patients with multifocal PTC.

**Results:**

Among the paediatric and adolescent patients, over a median follow-up time of 49 months, 133 had multifocal disease and 205 had unifocal disease. Multifocal PTC patients exhibited stronger invasiveness in the form of extrathyroidal extension, tumour diameter, lymph node metastasis, and distant metastasis. Multifocality (OR 2.68; *p* = 0.017), lateral lymph node metastasis (OR 2.85; *p* = 0.036), and distant metastasis (OR 4.28; *p* = 0.010) were identified as independent predictive factors for the recurrence/persistence of disease. Comparing the paediatric/adolescent vs. adult multifocal patients, the former demonstrated greater tumour invasiveness. Lateral lymph node metastasis (OR 6.36; *P* = 0.012) and distant metastasis (OR 3.70; *P* = 0.027) were independent predictive factors for recurrence/persistence of disease in multifocal patients, while age was not (OR 0.95; *P* = 0.455).

**Conclusion:**

Tumour multifocality independently predicts persistent/recurrent disease in paediatric and adolescent PTC patients.

## Introduction

Since 2000, thyroid cancer has become significantly more common in China, with approximately 22,000 new cases reported in 2022 [1]. Among children and adolescents, thyroid cancer is the most common endocrine cancer, and its incidence in this age group has been increasing in recent decades [2]. Papillary thyroid carcinoma (PTC) accounts for more than 90% of all paediatric cases, while follicular thyroid carcinoma (FTC) is less common. Medullary thyroid carcinoma (MTC), poorly differentiated tumours, and undifferentiated (anaplastic) thyroid carcinoma are rare in young patients [3]. In several case series, pediatric and adolescent PTC (aged ≤ 20 years, referred to thereafter as CA-PTC) seems to have a different clinicopathological profile and outcome [4]. In children and adolescents, PTC often exhibits more aggressive behaviour than in adults, with higher rates of extrathyroidal extension (ETE), lymph node metastasis (LNM), and distant metastasis, resulting in a greater risk of persistent/recurrent disease [3–8]. Therefore, it is crucial to identify independent clinical factors that can accurately predict outcomes and help clinicians tailor treatments and choose the appropriate level of follow-up.

PTC can manifest as a single unifocal tumour or multiple distinct lesions within the thyroid. The latter, termed multifocal PTC, has a prevalence ranging from 18 to 87% among PTC cases [9–11]. While some studies suggest an association between multifocality and PTC recurrence [5, 11–16], others indicate no such relationship [9, 16–18]. Multifocality has been observed in up to 43.3% of paediatric thyroid cancer cases [4]. In paediatric thyroid cancer patients, there is controversy surrounding the relationships between multifocality and recurrence and persistent disease [3, 5, 19–21].The guidelines for managing paediatric thyroid nodules and differentiated thyroid carcinoma, established by the American Thyroid Association (ATA) Pediatric Thyroid Cancer Guideline Taskforce, hold that multifocality does not influence recurrence [3], but in practice, multifocal PTC is often seen as a high-risk factor for disease progression, leading to more aggressive treatment and follow-up [9, 18]. The conflicting results on the prognostic value of multifocality in PTC, particularly in children, make PTC management highly challenging. Consequently, paediatric PTC patients may receive either excessive or inadequate treatment depending on how clinicians interpret the prognostic significance of multifocality in this population.

Due to the uncertainty about the prognostic value of multifocality in CA-PTC, this study sought to investigate its significance and prognostic implications. Our findings should offer insights into diagnostic and treatment strategies for young patients with multifocal PTC.

## Methods

### Study design

**Fig. 1:**
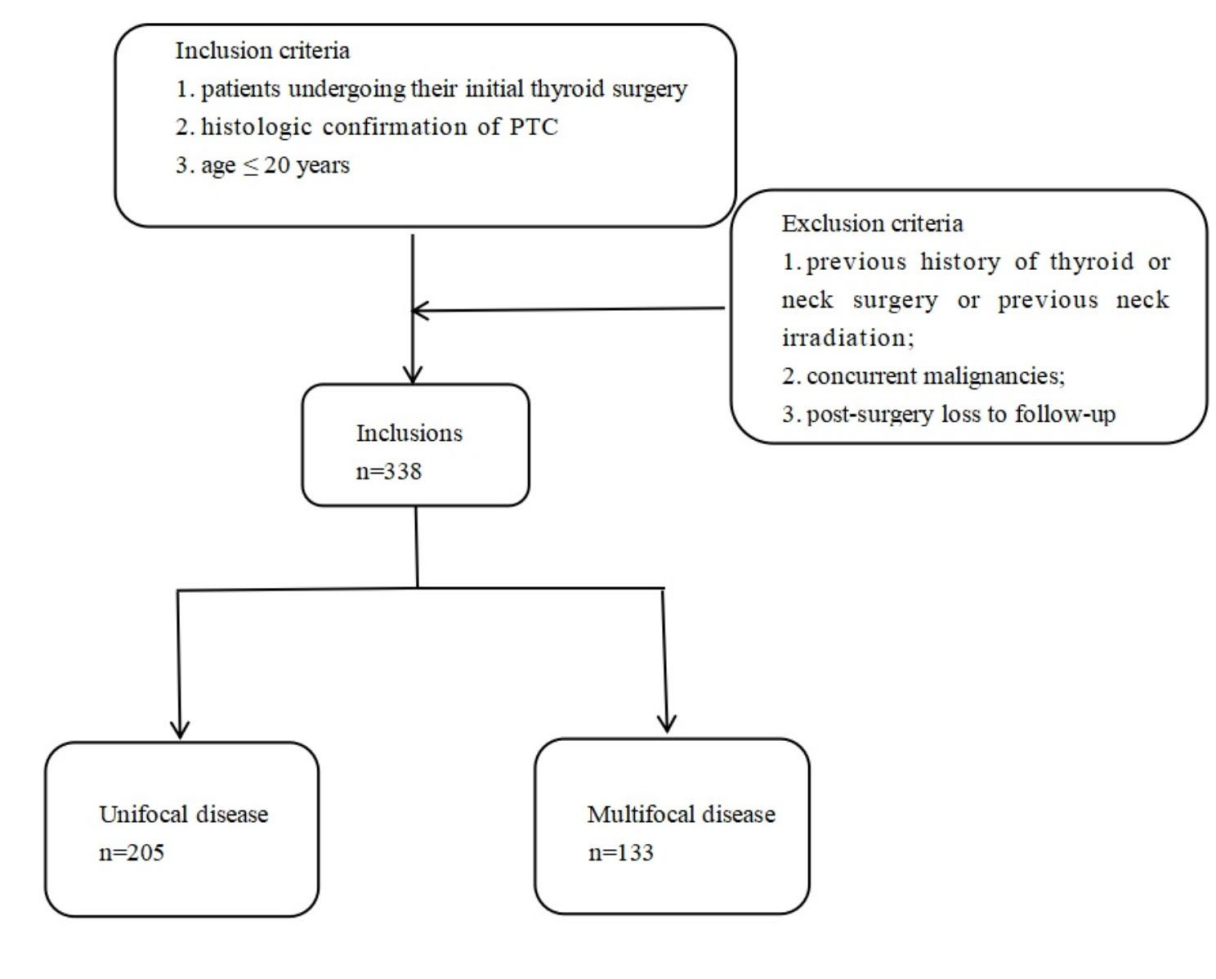
Inclusion and exclusion criteria

This study examined patients who underwent thyroidectomy for PTC at the Department of Thyroid Surgery, First Affiliated Hospital of Zhengzhou University, between May 1, 2012, and July 31, 2022. All patients underwent preoperative ultrasound conducted by two experienced ultrasound doctors. Decisions on the extent of surgery were at the discretion of the treating physician, with consideration for patient’s preference. The inclusion criteria were as follows: (1) patients who underwent initial thyroid surgery, (2) had a histologically confirmed PTC, and (3) age ≤ 20 years. The exclusion criteria: (1) previous history of thyroid or neck surgery or previous neck irradiation; (2) concurrent malignancies; and (3) loss to follow-up after surgery (Fig. 1). The median follow-up time was 49 months. The study included a total of 338 patients, among whom 133 had multifocal PTC. Based on the median follow-up time of multifocal CA-PTC patients, we included 114 PTC patients aged ≥ 20 years who underwent thyroid surgery at the Thyroid Surgery Department of the First Affiliated Hospital of Zhengzhou University during the same period. We included them to study the differences between adults and children with multifocal PTC. We compared the clinicopathological features of the unifocal and multifocal features of CA-PTC and the recurrent factors of CA-PTC; we also compared the clinical and pathological features of CA-PTC and adult PTC in multifocal patients and explored the recurrence factors in patients with multifocal PTC.

### Data collection

Patient demographic data, laboratory tests, imaging studies, and pathological findings were obtained from medical records. Patient demographic data included age, and sex. The collected clinical and pathological features included Hashimoto’s thyroiditis (HT), ETE, tumour diameter, central lymph node metastasis (CLNM), lateral lymph node metastasis (LLCM), distant metastasis, total lymph node (LN) harvesting, total LN involvement and radioactive iodine therapy (RAI) after surgery.

HT was diagnosed by postoperative sectioning and examination of paraffin-embedded thyroid tissue specimens. Serum antithyroglobulin and antithyroid peroxidase levels were measured within 30 days before surgery using the immuno-electrochemiluminescence method, and the patients were diagnosed with HT when these levels exceeded 115 IU/ml and 34 IU/ml, respectively. Bilateral disease and multifocal disease were considered together for statistical analysis [9]. Multifocality was defined as the presence of more than one lesion observed on neck colour Doppler ultrasound and confirmed pathologically as PTC. Tumour size was defined as the largest diameter of suspicious nodules on ultrasound. ETE, LNM, and the number of lymph nodes with metastases were determined through pathological examination. If no lymph node examination was performed, the patient was assumed to have no lymph node metastasis. ETE referred to breaking through the thyroid capsule and invading adjacent soft tissues, muscles, trachea, oesophagus, nerves or blood vessels. Distant metastasis was determined using single photon emission computed tomography (SPECT-CT) or computed tomography (CT) scans. Patients were staged according to the American Joint Committee on Cancer Staging System (8th edition).

Survival outcomes were determined by reviewing medical records and conducting telephone follow-up. Remission was defined as the absence of clinical or radiographic evidence of tumor post-initial treatment, with serum thyroglobulin < 1 ng/mL during thyroid-stimulating hormone (TSH) inhibition in total thyroid resection patients, and no detectable interfering antibodies. Tumor recurrence occurred if remission criteria were initially met but later evidence of locoregional disease or distant metastasis emerged via serology, imaging, or histology. Persistent disease referred to the failure to achieve remission criteria during the observation period.

### Statistical analysis

All the data analyses were conducted with the SPSS package version 25.0 (SPSS). Categorical variables are presented as counts and percentages, while continuous variables are described as means with their standard deviations (SDs) or medians and interquartile ranges (IQR). The chi-squared test or Fisher’s exact test was used to compare categorical variables between the unifocal and multifocal PTC groups. For continuous variables, the t test or nonparametric Mann‒Whitney U test was employed. Logistic regression models were used to assess the relationship between multifocality and disease recurrence/persistence. Kaplan–Meier curves and the log-rank test were used to analyse survival outcomes.

## Results

### Pathological characteristics in CA-PTC patients

A total of 338 patients (mean [SD] age, 16.4 [3.3] years; 237 women [70.1%]) were included in the study. Multifocal and unifocal patients accounted for 39.3% and 60.7% of the study population, respectively. Regarding tumor invasiveness characteristics, multifocal PTC patients, compared to unifocal PTC patients, exhibited an increased probability of extrathyroidal extension (ETE) (44.4% vs. 25.4%, *P* < 0.001), a longer primary tumour diameter (median [range], 2.4 [1.1–3.4] cm vs. 1.6 [0.9–2.7] cm, *P* = 0.005), a higher probability of lymph node metastasis (92.5% vs. 75.1%, *P* < 0.001), a higher probability of LLNM (74.4% vs. 29.3%, *P* < 0.001), a higher number of lymph nodes removed during surgery (median [range], 32.0 [19.0-48.5] vs. 8.0 [5.0–21.0], *P* < 0.001), a higher number of metastatic lymph nodes (median [range], 14.0 [7.0-19.5] vs. 3.0 [0.5-6.0], *P* < 0.001), a higher probability of distant metastasis (10.2% vs. 1.0%, *P* < 0.001). Moreover, patients with multifocal PTC had a higher probability of persistent/recurrent disease (24.1% vs. 5.4%, *P* < 0.001) (Table 1).

**Table 1:** The clinical characteristics and initial treatment of 338 CA-PTC patients

Characteristic	ALL PTC	Unifocal	Multifocal	*P*
Total patients	338	205	133	
Sex, n (%)				
Male	101 (29.9)	59 (28.8)	42 (31.6)	0.583
Female	237 (70.1)	146 (71.2)	91 (68.4)	
Age, mean ± SD	17.5 (15.0–19.0)	18.0 (15.5–19.0)	17.0 (14.0–19.0)	0.022
Hashimoto Thyroiditis, n (%)				
Absent	244 (72.2)	160 (78.0)	84 (63.2)	0.003
Present	94 (27.8)	45 (22.0)	49 (36.8)	
Extrathyroidal extension, n (%)				
Absence	227 (67.2)	153 (74.6)	74 (55.6)	< 0.001
Presence	111 (32.8)	52 (25.4)	59 (44.4)	
Primary tumor size				
median, (range), cm	1.8 (1.0-3.1)	1.6 (0.9–2.7)	2.4 (1.1–3.4)	0.005
≤2 cm, n (%)	196 (57.3)	129 (62.9)	65 (48.9)	0.032
2 to ≤ 4 cm, n (%)	111 (32.5)	59 (28.8)	50 (37.6)	
>4 cm, n (%)	35 (10.2)	17 (8.3)	18 (13.5)	
N stage, n (%)				
N0	61 (18.1)	51 (24.9)	10 (7.5)	< 0.001
N1a	118 (34.9)	94 (45.9)	24 (18.1)	
N1b	159 (47.0)	60 (29.3)	99 (74.4)	
Distant Metastases, n (%)				
M0	322 (95.3)	203 (99.0)	119 (89.5)	< 0.001
M1	16 (4.7)	2 (1.0)	14 (10.2)	
Total LN harvested, median, (range)	16.0 (6.8–35.0)	8.0 (5.0–21.0)	32.0 (19.0-48.5)	< 0.001
Total LN involved, median, (range)	5.5 (1.0–14.0)	3.0 (0.5-6.0)	14.0 (7.0-19.5)	< 0.001
RAI after surgery, n (%)				
Yes	177 (52.4)	62 (30.2)	99 (74.4)	< 0.001
No	161 (47.6)	143 (69.8)	34 (25.6)	
follow-up time, median (range), months	49.0 (29.0–72.0)	49.0 (29.0–74.0)	47.0 (29.0-70.5)	0.560
Persistent disease, n (%)				
Yes	11 (3.3)	1 (0.5)	10 (7.5)	< 0.001
No	327 (96.7)	204 (99.5)	123 (92.5)	
Recurrent disease, n (%)				
Yes	32 (9.5)	10 (4.9)	22 (16.5)	< 0.001
No	306 (90.5)	195 (95.1)	111 (83.5)	
persistent/recurrent disease.				
Yes	43(12.7)	11(5.4)	32(24.1)	< 0.001
No	295(87.3)	194(94.6)	101(75.9)	

### The relationship between multifocality and persistent/recurrent disease in CA-PTC patients

**Fig. 2:**
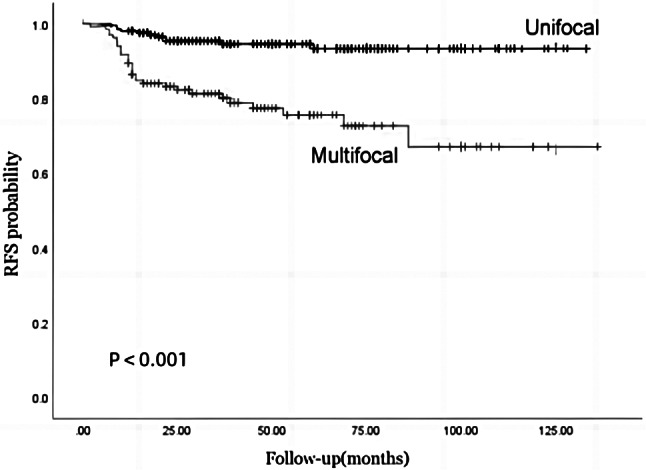
Kaplan‒Meier curves estimating the recurrence-free survival (RFS) of CA-PTC patients with multifocal versus unifocal disease

CA-PTC patients had a median follow-up time of 49 months. There were 43 patients (12.7%) with persistent/recurrent disease, including 11 (5.4%) with unifocal disease and 32 (24.1%) with multifocal disease (Table 1). During the follow-up period, multifocal patients had a higher probability of persistent/recurrent disease compared to unifocal patients (24.1% vs. 5.4%, *P* < 0.001). (Table 1) Univariate analysis found that age (OR 0.89; *P* = 0.009), multifocality (OR 5.59; *P* < 0.001), CLNM (OR 10.72; *P* = 0.020), LLNM (OR 7.19; *P* < 0.001), and distant metastasis (OR 10.89 *P* < 0.001) were significantly associated with persistent/recurrent disease (Table 2). Regarding persistent/recurrent disease, multivariate analysis found that multifocality (OR 2.68; *P* = 0.017), LLNM (OR 2.85; *P* = 0.036), and distant metastasis (OR 4.28; *P* = 0.010) remained independent variables for predicting persistent/recurrent disease. (Table 2). Fig 2 represented the Kaplan-Meier curves estimating the recurrence and survival according to the multifocality of CA-PTC. Compared to patients with unifocal disease, patients with multifocal disease had significantly lower disease-free survival (*P*<0.001).

**Table 2:** Univariate and multivariate analysis of predictive factors for persistent/recurrent disease in CA-PTC

	OR (95% CI)	*P*	Adjusted OR (95% CI)	*P*
Sex, men	0.68 (0.32–1.44)	0.312	-	
Age	0.89 (0.81–0.97)	0.009	0.95 (0.86–1.05)	0.327
Multifocality	5.59 (2.70-11.55)	< 0.001	2.68 (1.20–6.03)	0.017
Hashimoto Thyroiditis	0.88 (0.42–1.82)	0.727	-	
Extrathyroidal extension	1.25 (0.64–2.43)	0.514	-	
Primary tumor size	1.21 (0.97–1.51)	0.097	-	
Primary tumor size >4 cm	1.49 (0.58–3.82)	0.410	-	
Central Lymph Node Metastases	10.7 (1.45–79.50)	0.020	2.96 (0.35–25.36)	0.322
Lateral lymph node metastasis	7.19 (3.10-16.69)	< 0.001	2.85 (1.07–7.57)	0.036
Distant metastasis	10.8 (3.81–31.11)	< 0.001	4.28 (1.42-12.90)	0.010

### Comparison of clinical and pathological characteristics between paediatric and adult patients with multifocal PTC

We added 114 patients aged over 20 years with multifocal PTC as an adult comparison group. The CA-PTC patients had a higher proportion of females than the adult PTC cohort (86.0% vs. 68.4%, *P* = 0.001) (Table 3). CA-PTC patients had a higher prevalence of HT (36.8% vs. 21.1%, *P* = 0.007)(Table 3). Regarding tumour invasiveness characteristics, CA-PTC had a higher probability of ETE (44.4% vs. 28.9%, *P* = 0.012), a larger primary tumour size (median [range], 2.4 [1.1–3.4] cm vs. 0.8 [0.5–1.5] cm, *P* < 0.001), and higher probabilities of lymph node metastasis (92.5% vs. 40.4%, *P* < 0.001), LLNM (74.4% vs. 17.5%, *P* < 0.001), and distant metastasis (10.2% vs. 0%, *P* < 0.001)(Table 3).

**Table 3:** Clinical characteristics and initial treatment of 247 multifocal PTC patients

Characteristic	ALL age	>20	≤ 20	*P*
Total patients	247	114	133	
Sex, n (%)				
Male	58 (23.5)	16 (14.0)	42 (31.6)	0.001
Female	189 (76.5)	98 (86.0)	91 (68.4)	
Age, median, (range)	20.0 (17.0–49.0)	50.0 (41.0–55.0)	17.0 (14.0–19.0)	< 0.001
Hashimoto Thyroiditis, n (%)				
Absent	174 (70.4)	90 (78.9)	84 (63.2)	0.007
Present	73 (29.6)	24 (21.1)	49 (36.8)	
Extrathyroidal extension, n (%)				
Absence	155 (62.8)	81 (71.1)	74 (55.6)	0.012
Presence	92 (37.2)	33 (28.9)	59 (44.4)	
Primary tumor size				
Median, (range), cm	1.3 (0.7–2.6)	0.8 (0.5–1.5)	2.4 (1.1–3.4)	< 0.001
≤2 cm, n (%)	169 (68.4)	104 (91.2)	65 (48.9)	< 0.001
2 to ≤ 4 cm, n (%)	58 (23.5)	8 (7.0)	50 (37.6)	
>4 cm, n (%)	20 (8.1)	2 (1.8)	18 (13.5)	
N stage, n (%)				
N0	78 (31.6)	68 (59.6)	10 (7.5)	< 0.001
N1a	50 (20.2)	26 (22.8)	24 (18.1)	
N1b	119 (48.2)	20 (17.5)	99 (74.4)	
Distant Metastases, n (%)				
M0	233 (94.3)	114 (100.0)	119 (89.5)	< 0.001
M1	14 (5.7)	0 (0)	14 (10.2)	
RAI after surgery, n (%)				
Yes	142 (57.5)	43 (37.7)	99 (74.4)	< 0.001
No	105 (42.5)	71 (62.3)	34 (25.6)	
Persistent disease, n (%) Yes No	10 (4.0) 237 (96.0)	0 (0) 114 (100)	10 (7.5) 123 (92.5)	0.003
Recurrent disease, n (%)				
Yes	28 (11.3)	6 (5.3)	22 (16.5)	0.005
No	219 (88.7)	108 (94.7)	111 (83.5)	
Persistent/recurrent disease, n (%) Yes No	38 (15.4) 209 (84.6)	6 (5.3) 108 (94.7)	32 (24.1) 101 (75.9)	< 0.001

### The relationship between age and persistent/recurrent disease in paediatric vs. adult patients with multifocal PTC

Among the multifocal patients, 38 (15.4%) developed persistent/recurrent disease, including 6 (5.3%) with adult PTC and 32 (24.1%) with CA-PTC (Table 3). During follow-up, CA-PTC patients had a higher probability of persistent/recurrent disease than adult patients with multifocal PTC (24.1% vs. 5.3%, *P* < 0.001). Univariate analysis showed that age (> 20) (OR 0.18; *p* < 0.001), primary tumour size (OR 1.46; *p* = 0.002), CLNM (OR 10.50; *p* = 0.001), LLNM (OR 12.40; *p* < 0.001), and distant metastasis (OR 9.02; *p* < 0.001) were significantly associated with persistent/recurrent disease (Table 4). With respect to persistent/recurrent disease, a multivariate analysis found that LLNM (OR 6.36; *p* = 0.012) and distant metastasis (OR 3.70; *p* = 0.027) remained independent variables for predicting persistent/recurrent disease, while age was not a significant factor (OR 0.95; *P* = 0.327) (Table 4).

**Table 4:** Univariate and multivariate analysis of predictive factors for persistent/recurrent disease in multifocal PTC patients

	OR (95% CI)	*P*	Adjusted OR (95% CI)	*P*
Sex, man	1.01 (0.45–2.29)	0.974	-	
Age,>20	0.18 (0.70 − 0.44)	< 0.001	0.65 (0.21–2.03)	0.455
Hashimoto Thyroiditis	0.83 (0.38–1.81)	0.635	-	
Extrathyroidal extension	1.64 (0.82–3.30)	0.163	-	
Primary tumor size	1.46 (1.15–1.85)	0.002	1.03 (0.77–1.37)	0.861
Primary tumor size >4 cm	1.96 (0.67–5.76)	0.221	-	
Central Lymph Node Metastases	10.50 (2.46–44.82)	0.001	1.52 (0.22–10.75)	0.674
Lateral lymph node metastasis	12.40 (4.24–36.23)	< 0.001	6.36 (1.50-26.96)	0.012
Distant metastasis	9.02 (2.93–27.81)	< 0.001	3.70 (1.16–11.84)	0.027

## Discussion

Risk stratification plays a critical role in customizing the management of thyroid cancer. Initial risk assessment helps guide various clinical management decisions that need to be made during initial diagnosis and treatment, and can be used to guide early monitoring and treatment management decisions [9, 22]. Compared with adults, patients with CA-PTC often experience more persistent or recurrent disease, in contrast to adults [3, 4, 8]. Hence, accurate risk stratification is vital for guiding treatment and follow-up intensity in the paediatric population. There is no well-established system for predicting the prognosis in of CA-PTC patients. The TNM classification system has limitations in predicting prognosis in children [3]. Although numerous studies have sought prognostic and recurrence factors in CA-PTC, the link between multifocality and prognosis in thyroid cancer patients, particularly children, remains contentious [3, 13, 15, 16, 18, 19, 21, 23]. In this cohort study, we compared the clinical and pathological characteristics of CA-PTC patients with multifocal and unifocal presentations. We identified multifocal tumours as a predictive factor for persistent/recurrent disease in CA-PTC patients.

Early studies showed that tumour multifocality increases the risk of recurrence [11–16, 23]. A single-centre studyconducted by Woo Ri Choi et al. in South Korea, in 2390 patients (37.3% of whom had multifocal PTC), found a significant association between multifocality and an adjusted hazard ratio (HR) for recurrence-free survival (RFS) of 1.93 (95% confidence interval = 1.33–2.80; *P* = 0.001) [12]. A systematic review and meta-analysis conducted by Hyeonkyeong Kim et al., encompassing 26 studies and 33,976 patients, highlighted that multifocal PTC patients exhibited a significantly higher recurrence rate than unifocal PTC patients (pooled HR, 1.81; 95% CI, 1.52–2.14) [23]. . In CA-PTC, a study by Hyung Kwon Byeon et al. indicated a correlation between multifocality and recurrence in paediatric PTC patients (HR 19.388; 95% CI 2.739-137.245) [19], which aligns with our findings. However, recent research has also indicated that tumour multifocality is not associated with recurrence [9, 16–18]. A study by Yossi et al., which included 1,039 patients and utilized statistical methods with propensity score matching, found that multifocality of PTC was not an independent prognostic factor for disease persistence/recurrence [9]. In CA-PTC patients, a study by Jiaying Chen et al. from China suggested that recurrence in paediatric PTC patients was related only to lymphovascular invasion and is unrelated to multifocality [20]. The inconsistency in these findings might be related to the low recurrence rate of PTC, but the specific reasons need to be further explained.

Our study found that multifocality in CA-PTC patients was more aggressive than unifocality, consistent with prior investigations [10, 11, 13–16, 18, 23]. In CA-PTC, multifocality, lateral neck lymph node metastasis, and distant metastasis were significantly linked to persistent/recurrent disease. This indicates that more aggressive treatment measures and more intense follow-up are needed for patients with multifocal PTC in CA-PTC. Additionally, multifocal patients tended to be younger and to have HT more often. When comparing multifocal CA-PTC patients with multifocal adult patients, our study confirmed the greater aggressiveness of CA-PTC [2–5, 7, 8, 15], even within multifocal tumours. Furthermore, in multifocal PTC patients, lateral neck lymph node metastasis and distant metastasis independently predicted persistent/recurrent disease, age showing no significant association.

In the comparison between paediatric and adult patients with multifocal PTC, our study confirmed the previous findings that paediatric PTC displays more aggressive characteristics [2–5, 7, 8, 15]. Age was not an independent predictor of persistent or recurrent disease in patients with multifocal PTC, consistent with some earlier studies [3, 4, 8]. Importantly, this observation pertains specifically to multifocal PTC within our study. These findings suggests that in multifocal PTC patients, treatment strategies and postoperative management for paediatric and adolescent patients can be guided by the ATA guidelines established for adult patients.

Our study has limitations. First, it is a retrospective investigation, that is susceptible to selection bias and recall bias. Furthermore, all study participants were drawn from the same hospital without external validation. A larger sample will be needed to minimize this bias. Additionally, there may be selection bias in comparing paediatric and adult patients with multifocal PTC, given that we included only adult patients who underwent thyroid surgery at the Department of Thyroid Surgery, the First Affiliated Hospital of Zhengzhou University, within one month. Finally, the follow-up was relatively short, lasting a median follow-up time of 49 months. Hence, our findings should be interpreted carefully.

## Conclusions

Multifocal CA-PTC presented more aggressive characteristics than unifocality PTC. Multifocality was linked to postoperative persistent/recurrent disease in CA-PTC. Within the group of multifocal PTC patients, CA-PTC displayed more aggressive characteristics than adult PTC, yet age did not correlate with persistent/recurrent disease in multifocal PTC patients.

## Data Availability

The datasets used and/or analysed during the current study are available from the corresponding author on reasonable request.

## Abbreviations

PTC: Papillary Thyroid Carcinoma
FTC: Follicular Thyroid Carcinoma
MTC: Medullary Thyroid Carcinoma
CA-PTC: Pediatric and Adolescent PTC
ETE: Extrathyroidal Extension
LNM: Lymph Node Metastasis
ATA: The American Thyroid Association
HT: Hashimoto’s Thyroiditis
CLNM: Central Lymph Node Metastasis
LLNM: Lateral Lymph Node Metastasis
LN: Lymph Node
RAI: Radioactive Iodine Therapy
